# Understanding the role of obesity in endocrine therapy for postmenopausal breast cancer: significance of the BIG 1–98 and ATAC trial data

**DOI:** 10.1007/s12672-025-02857-w

**Published:** 2025-07-01

**Authors:** Sixten Harborg, Signe Borgquist

**Affiliations:** 1 Department of Oncology, Aarhus University, Aarhus University Hospital, Palle Juul-Jensen Boulevard 99, 8200 Aarhus N, Denmark; 2 Department of Clinical Epidemiology, Aarhus University, Aarhus University Hospital, Aarhus, Denmark; 3 Department of Nutrition, Harvard T.H. Chan School of Public Health, Boston, MA USA; 4 Department of Oncology, Clinical Sciences, Lund University, Lund, Sweden

## Abstract

Obesity is a known risk factor for poor breast cancer outcomes, but its impact on endocrine therapy efficacy remains unclear. While the ATAC trial suggests reduced effectiveness of aromatase inhibitors (AIs) in women with obesity, the BIG 1–98 trial found no significant differences. Clinical and biological evidence indicates that obesity may impair AI efficacy by increasing aromatase activity and altering drug metabolism, whereas tamoxifen remains largely unaffected. Pooling data from these trials would enable detailed analyses across body composition categories, addressing discrepancies and potentially guide personalized treatment strategies. Understanding the interaction between obesity and endocrine therapy is crucial for optimizing breast cancer care.

## Introduction

Breast cancer remains a global health challenge, with over two million new cases annually [1]. Among postmenopausal women, hormone receptor-positive (HR+) breast cancer is the most common subtype, accounting for a significant proportion of cases. Endocrine therapies, particularly tamoxifen and aromatase inhibitors (AIs), have revolutionized treatment and improved survival rates. However, treatment outcomes vary widely, with obesity emerging as a critical factor influencing prognosis. Obesity, a growing epidemic worldwide, affects nearly 40% of postmenopausal women in high-income countries, making its impact on breast cancer outcomes an urgent research priority [2]. 

Obesity and estrogen regulation are interrelated and therefore the efficacy of endocrine therapies is hypothesized to differ depending on body composition and type of endocrine therapy. The large-scaled, randomized trials Breast International Group (BIG) 1–98 [3] and Arimidex, Tamoxifen, Alone or in Combination (ATAC) [4] are pivotal studies comparing the efficacy of tamoxifen and AIs, offering invaluable insights into the treatment of early HR+ breast cancer. Despite their significant clinical contributions, neither trial is designed to fully address the complex interplay between obesity and endocrine therapy outcomes. Previous retrospective explanatory studies of the two trials have emphasized that obesity may negatively influence the effectiveness of AIs, necessitating further exploration of this relationship [5, 6]. However, the results remain inconclusive as the BIG 1–98 trial reported a similar efficacy of AIs across baseline body mass index groups (BMI), while the ATAC trial reported that the clinical benefit of AIs was largely attenuated with higher degrees of baseline BMI. Combining data from ATAC and BIG 1–98 presents a unique opportunity to unravel this complexity, potentially guiding more effective and personalized treatment strategies. This commentary underscores the necessity of combining these two datasets to investigate whether the efficacy of AIs is compromised in women with obesity compared to tamoxifen efficacy in the same population, an insight potentially impacting survival outcomes.

## Current evidence on obesity and endocrine therapy outcomes

Obesity is a well-established risk factor for worse outcomes in breast cancer and is associated with a 35–40% increased risk of recurrence and mortality [7]. The ATAC trial demonstrated that women with obesity receiving the AI anastrozole had higher recurrence rates than their normal-weight counterparts (HR 1.53, 95% CI 1.06–2.21). Conversely, tamoxifen’s efficacy appeared unaffected by BMI, suggesting a differential impact of obesity across these two types of endocrine therapies [5]. Meanwhile, the BIG 1–98 trial, which compared tamoxifen and another AI – letrozole, reported no significant BMI-dependent differences between the two endocrine therapies in efficacy [6]. Both trials adjusted for key prognostic tumor characteristics such as tumor grade and nodal involvement, indicating that the observed differences are unlikely to be solely explained by baseline disease severity However, inconsistent BMI categorizations and reporting from the two trials complicate direct comparisons.

Existing research underscores the limitations of individual trials in capturing the full scope of obesity’s impact on cancer outcomes [8, 9]. Real-world studies have tried to address how obesity impact breast cancer outcomes in AI-treated patients, reporting similar recurrence rates as the ATAC-trial but fail to establish a comparator treatment (i.e. tamoxifen) [8]. Pooling data from the BIG 1–98 and ATAC trials would overcome these limitations, enabling robust analyses of obesity’s role in endocrine therapy. This approach would enable subgroup analyses across BMI categories, helping clarify the interplay between obesity, type of endocrine therapy, and breast cancer outcomes.

### Biological mechanisms linking obesity and endocrine therapy

**Fig. 1:**
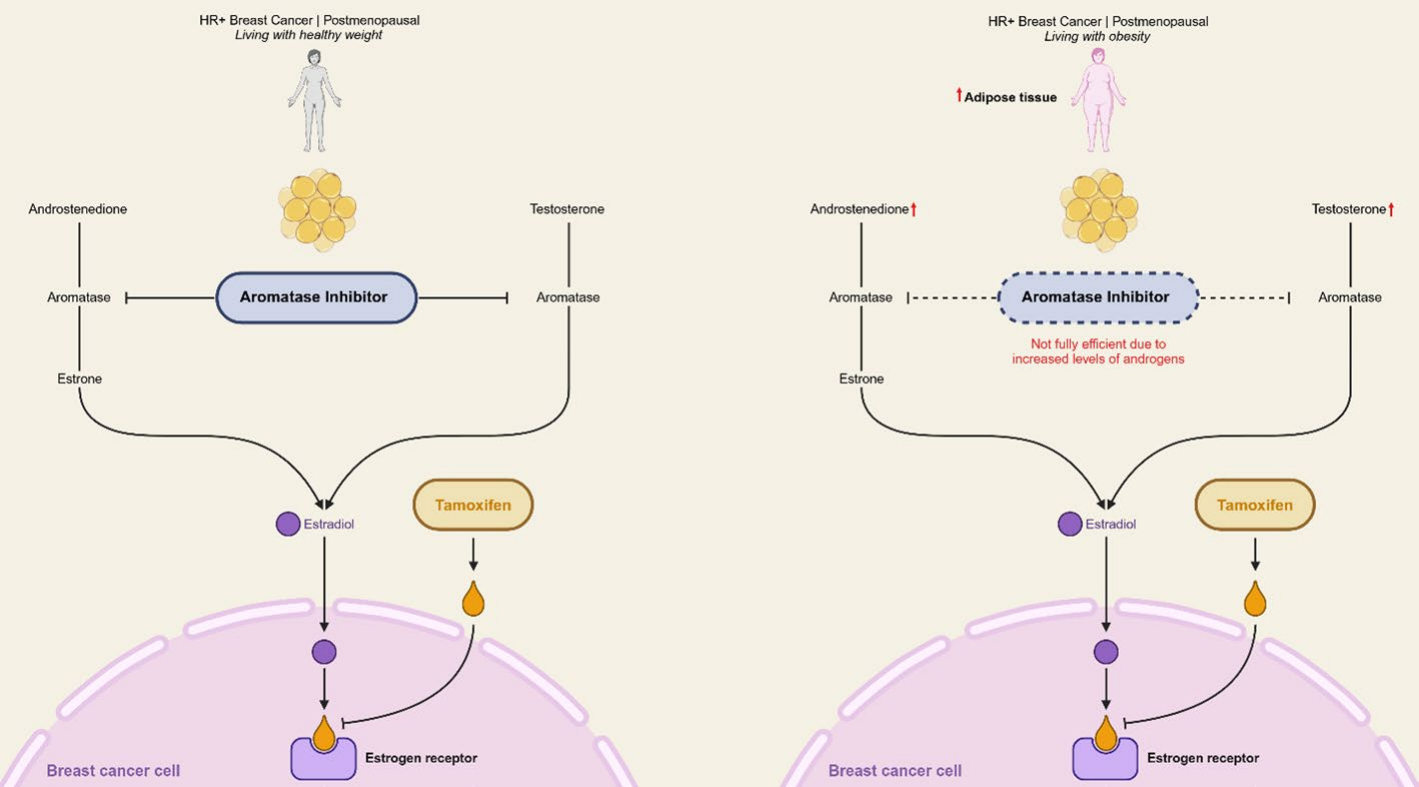
Impact of Obesity on Aromatase Inhibitor Efficacy in Postmenopausal Hormone Receptor-positive Breast Cancer. * Increased adipose tissue leads to higher androgen levels, which may interfere with aromatase inhibitor efficacy, reducing its ability to suppress estrogen synthesis (dashed inhibition line). As a result, residual estradiol can still activate the estrogen receptor. In contrast, tamoxifen remains effective in blocking the receptor, regardless of body composition. Figure created by the authors using BioRender *(*www.biorender.com*).*

The biological mechanisms by which obesity impacts endocrine therapy outcomes are multifaceted and rooted in the physiological role of adipose tissue. AIs function by inhibiting aromatase, the enzyme responsible for converting androgens into estrogens in adipose tissue [10]. In individuals with obesity, increased adipose tissue leads to elevated aromatase activity and residual estrogen production, potentially undermining AI efficacy (Fig. 1) [10]. Tamoxifen, which directly blocks estrogen receptors, may be less affected by variations in circulating estrogen levels, potentially explaining its consistent efficacy across BMI categories [5, 11]. 

Pharmacodynamic variability in efficacy across the different AIs is another critical factor. Letrozole achieves 99.1% aromatase inhibition versus 97.3% for anastrozole in standard doses, which possibly could explain the reported discrepancies from the trials [12]. However, obesity is known to alter AI distribution and metabolism; women with obesity exhibit 35% lower plasma letrozole concentrations per unit BMI (*r*=− 0.35, *p* = 0.02), potentially comprising therapeutic thresholds [13]. Residual estradiol levels under AI therapy correlate positively with BMI (*r* = 0.41, *p* = 0.01), with patients with obesity maintaining median levels of 12.5 pg/mL versus 9.0 pg/mL in patients without obesity [13]. Mechanistic studies have uncovered that in obesity, hypertrophic adipocytes exhibit upregulated aromatase expression, leading to 2–3-fold higher intratumoral estrogen levels compared to lean individuals. Similarly preclinical models demonstrate that diet-induced obesity in mice reduces letrozole sensitivity, with tumors in obese mice showing 30% less growth suppression than lean countrerparts [14, 15]. These findings complement the clinical observations and highlights the need for investigation into individualized treatment approaches based on BMI and metabolic profiles [16]. 

## Why combine data from BIG 1–98 and ATAC?

The BIG 1–98 (*N* = 8010) and ATAC (*N* = 9366) trials represent complementary datasets, with overlapping objectives, methodologies, and study periods that make their integration particularly valuable. Combining data from these trials provides an unprecedented opportunity to address critical gaps in our understanding of obesity’s impact on endocrine therapy outcomes. A larger pooled cohort would enable more granular subgroup analyses, including underrepresented BMI categories, such as severe obesity. Pooling these datasets would also allow for a deeper exploration of dose-response relationships, particularly regarding the thresholds of AI efficacy across varying BMI groups. The integration of these datasets could clarify whether the discrepancies observed in the individual trials are due to methodological differences or genuine variations in treatment efficacy.

Such combined analyses could also inspire research into the potential development of personalized dosing strategies, such as adjusting AI doses based on body surface area, weight, or circulating levels of estradiol to improve clinical outcomes in patients with obesity.

While pooling data from ATAC and BIG 1–98 offers a unique opportunity to enhance statistical power and resolution, such efforts must carefully account for inherent differences between the trials—including the type of AI administered (anastrozole vs. letrozole), study design, and adherence patterns. Moreover, obesity-related side effects such as AI-induced arthralgia may differentially affect compliance, further complicating the interpretation of efficacy estimates across BMI groups. Rather than undermining the rationale, these complexities underscore the necessity of conducting a carefully harmonized, individual patient data meta-analysis to resolve inconsistencies and derive robust clinical insights.

## Clinical and research implications

The findings from the proposed combined analyses of the BIG 1–98 and ATAC trial datasets could have profound implications for clinical practice and research. If AIs are shown to be less effective in women with obesity, this would necessitate a re-evaluation of current treatment guidelines. Tailoring endocrine therapy based on BMI and associated biomarkers could possibly optimize clinical outcomes for this subgroup of patients. For example, higher dosing of AIs might mitigate the incomplete estrogen suppression observed in patients with obesity, although clinical trials are needed to validate this approach [17]. 

Incorporating BMI-specific guidelines into clinical practice could ensure equitable care for all patients independent of body composition. Stratifying patients by BMI during treatment planning would identify those who might benefit from modified dosing or additional interventions. The molecular basis of obesity-related resistance to AIs could also pave the way for novel therapeutic strategies in breast cancer, such as selective cytokine inhibitors, insulin-sensitizing agents or weight loss drugs.

Future research should prioritize the development of predictive biomarkers for AI response in the context of obesity. The availability of large-scale, combined datasets from the BIG 1–98 and ATAC trials would accelerate these efforts, offering valuable insights into both individual and population-level trends. Such efforts would bridge the gap between clinical research and real-world applications, ensuring that findings translate into tangible benefits for patients.

## Conclusion

The interplay between obesity and adjuvant endocrine therapy outcomes is a critical yet underexplored area in clinical breast cancer research. The BIG 1–98 and ATAC trials provide a unique opportunity to address this gap through combined analyses. By integrating these datasets, clinical researchers can gain definitive insights into whether women with obesity derive less benefit from AIs compared to tamoxifen, ultimately guiding more effective and personalized treatment strategies. Given the increasing global prevalence of obesity, addressing its implications in breast cancer treatment is a public health imperative. Collaborative efforts to analyze and expand these key datasets will be pivotal to settling the question about potential survival disparities among postmenopausal women with HR+ breast cancer that are living with obesity.

## Data Availability

No datasets were generated or analysed during the current study.
